# Recent advances in phlebotomine sand fly research related to leishmaniasis control

**DOI:** 10.1186/s13071-015-0712-x

**Published:** 2015-02-27

**Authors:** Paul A Bates, Jerôme Depaquit, Eunice AB Galati, Shaden Kamhawi, Michele Maroli, Mary Ann McDowell, Albert Picado, Paul D Ready, O Daniel Salomón, Jeffrey J Shaw, Yara M Traub-Csekö, Alon Warburg

**Affiliations:** Division of Biomedical and Life Sciences, Faculty of Health and Medicine, Lancaster University, Lancaster, UK; Université de Reims Champagne-Ardenne, ANSES, EA4688 – USC [Transmission vectorielle et épidémiosurveillance de maladies parasitaires (VECPAR)], 51, rue Cognacq-Jay, 51096 Reims Cedex, France; Departamento de Epidemiologia, Faculdade de Saúde Pública, Universidade de São Paulo, SP 01246-904 São Paulo, Brazil; Vector Molecular Biology Section, Laboratory of Malaria and Vector Research, National Institute of Allergy and Infectious Diseases, National Institutes of Health, 12735 Twinbrook Parkway, Rockville, MD 20852 USA; c/o Istituto Superiore di Sanità, Rome, Italy; Eck Institute for Global Health, Department of Biological Sciences, University of Notre Dame, Notre Dame IN, USA; ISGlobal, Barcelona Ctr. Int. Health Res. (CRESIB), Hospital Clínic, Universitat de Barcelona, Barcelona, E-08036 Spain; Department of Disease Control, Faculty of Infectious and Tropical Diseases, London School of Hygiene and Tropical Medicine, Keppel Street, London, WC1E 7HT UK; National Institute of Tropical Medicine-MOH, CONICET, Neuquen y Jujuy s/n, 3370, Puerto Iguazu, Argentina; Biomedical Sciences Institute, Universidade de São Paulo, SP, São Paulo, Brazil; Laboratório de Biologia Molecular de Parasitas e Vetores, Instituto Oswaldo Cruz, Fiocruz, Rio de Janeiro, RJ, Brazil; Kuvin Center for the study of Infectious & Tropical Diseases, Institute of Medical Research Israel-Canada/ Faculty of Medicine, Hebrew University of Jerusalem, Ein Kerem, Jerusalem, 91120 Israel

**Keywords:** Phlebotomine sand flies, Human leishmaniasis, Vector control, Leishmaniasis control, ISOPS

## Abstract

Phlebotomine sand flies are the subject of much research because of the role of their females as the only proven natural vectors of *Leishmania* species, the parasitic protozoans that are the causative agents of the neglected tropical disease leishmaniasis. Activity in this field was highlighted by the eighth International Symposium on Phlebotomine Sand flies (ISOPS) held in September 2014, which prompted this review focusing on vector control. Topics reviewed include: Taxonomy and phylogenetics, Vector competence, Genetics, genomics and transcriptomics, Eco-epidemiology, and Vector control. Research on sand flies as leishmaniasis vectors has revealed a diverse array of zoonotic and anthroponotic transmission cycles, mostly in subtropical and tropical regions of Africa, Asia and Latin America, but also in Mediterranean Europe. The challenge is to progress beyond descriptive eco-epidemiology, in order to separate vectors of biomedical importance from the sand fly species that are competent vectors but lack the vectorial capacity to cause much human disease. Transmission modelling is required to identify the vectors that are a public health priority, the ones that must be controlled as part of the integrated control of leishmaniasis. Effective modelling of transmission will require the use of entomological indices more precise than those usually reported in the leishmaniasis literature.

## Introduction

Phlebotomine sand flies (Diptera: Psychodidae, Phlebotominae) are the subject of far more research [1,2] than might be expected based on the relatively small size of this insect subfamily. This arises from the role of their females as the only proven natural vectors of *Leishmania* species (Kinetoplastida: Trypanosomatidae), the parasitic protozoans that are the causative agents of the neglected tropical disease leishmaniasis [3] (Table 1). Activity in this research field was highlighted by the eighth International Symposium on Phlebotomine Sand flies (ISOPS VIII; http://www.isops8.org/en/libro-de-resumenes-2/), which was held in Puerto Iguazu, Argentina, in September 2014, thanks to the local organizing committee (led by ODS) and support from WHO, PAHO and others. ISOPS IX will be held in Reims, France, in June-July 2016 (organized by JD and colleagues; http://www.isops9.org).

ISOPS VIII included 241 presentations by authors from 35 countries, and most of the reports related to leishmaniasis and its control. This level of interest prompted the current review, which complements recent reviews on sand fly biology [1,2] by focusing on leishmaniasis control.

**Table 1:** **Generic (subgeneric) classification of phlebotomine sand flies focusing on the main vectors of** ***Leishmania*** **species causing most human leishmaniasis**

**Six long- accepted genera (a)**	**Distribution of genera (a)**	**Main vectors of human leishmaniasis (a), in genus of first column and aligned with alternative classification in fourth column**	**Alternative genus (subgenus) classification in Latin American, Francophone and other countries (b)**	**Form of human leishmaniasis,** ***Leishmania*** **parasites transmitted (c)**
*Phlebotomus*	Europe	*P. (Larroussius)* spp.	None	ZVL, *Le. (Le.) infantum infantum*
		*P. (Paraphlebotomus) sergenti*	None	ACL or ZCL, *Le. (Le.) tropica*
	Africa	*P. (Larroussius)* spp.	None	ZVL, *Le. (Le.) i. infantum*
		*P. (Larroussius)* spp.	None	AVL or ZVL, *Le. (Le.) donovani*
		*P. (Synphlebotomus)* spp.	None	AVL or ZVL, *Le. (Le.) donovani*
		*P. (Paraphlebotomus) sergenti*	None	ACL or ZCL, *Le. (Le.) tropica*
		*P. (Phlebotomus) papatasi*	None	ZCL, *Le. (Le.) major*
		*P. (Phlebotomus) duboscqi*	None	ZCL, *Le. (Le.) major*
	Asia	*P. (Larroussius)* spp.	None	ZVL, *Le. (Le.) i. infantum*
		*P. (Euphlebotomus) argentipes*	None	AVL, *Le. (Le.) donovani*
		*P. (Paraphlebotomus) sergenti*	None	ACL & ZCL, *Le. (Le.) tropica*
		*P. (Adlerius)* spp.	None	ZCL, *Le. (Le.) tropica*
		*P. (Phlebotomus) papatasi*	None	ZCL, *Le. (Le.) major*
	Australia, Asia	No incriminated vectors	*Australophlebotomus*	Not applicable
	Asia, Australia	No incriminated vectors	*Idiophlebotomus* (H)	Not applicable
	Africa	No incriminated vectors	*Spelaeophlebotomus* (H)	Not applicable
*Sergentomyia*	Europe, Africa, Asia, Oceania, Australia	No incriminated vectors	None of 6+ subgenera of *Sergentomyia*	Not applicable
	Africa, Asia	No incriminated vectors	*Grassomyia, Parvidens, Spelaeomyia*	Not applicable
*Chinius* (H)	Asia	No incriminated vectors	None	Not applicable
*Warileya*	C & S America	No incriminated vectors	*Warileya* (H), *Hertigia* (H)	Not applicable
*Brumptomyia*	C & S America	No incriminated vectors	None	Not applicable
*Lutzomyia*	C & S America	*L. (Lutzomyia) longipalpis s.l.*	None	ZVL, *Le. (Le.) infantum chagasi*
	S America	*L. (Pifanomyia) evansi*	*Pintomyia (Pifanomyia)*	ZVL, *Le. (Le.) i. chagasi*
	C & S America	*L. (Nyssomyia) olmeca*	*Bichromomyia*	ZCL, *Le. (Le.) mexicana s.l.*
	S America	*L. (Nyssomyia) flaviscutellata*	*Bichromomyia*	ZCL, *Le. (Le.) amazonensis*
	S America	*L. (Helcocyrtomyia)* spp.	None	ZCL, *Le. (Le.) mexicana s.l.*
	S America	*L. (Nyssomyia)* spp., *L. (Pifanomyia)* spp., *L. (Psychodopygus)* spp.	*Nyssomyia, Pintomyia (Pifanomyia), Psychodopygus*	ZCL & MCL, *Le. (Vi.) braziliensis*
	S America	*L. (Helcocyrtomyia)* spp.	None	ZCL & MCL, *Le. (Vi.) peruviana*
	C & S America	*L. (Nyssomyia)* spp.	*Nyssomyia*	ZCL, *Le. (Vi.) panamensis, Le. (Vi.) guyanensis*
	Americas	Suspected vectors in one or more of the 26 subgeneric groups of *Lutzomyia*	One or more of the 31 subgenera classified in 18 genera	American parasites (above); plus *Le. (Le.) i. infantum* in North American foxhounds

(a) Lewis et al. [8]; Young and Duncan [9]; Ready [1]; Maroli et al. [2]. (b) Galati [4,5]; Rispail and Léger [6]. (c) Shaw [29]; WHO [46]; Ready [1]; Maroli et al. [2]; ACL: anthroponotic cutaneous leishmaniasis; ZCL; zoonotic cutaneous leishmaniasis; MCL: muco-cutaneous leishmaniasis; AVL: anthroponotic visceral leishmaniasis; ZVL; zoonotic visceral leishmaniasis; (H) The five genera classified in tribe Hertigiini by Galati [4,5], who placed the rest in tribe Phlebotomini.

## Review

### Taxonomy and phylogenetics

In the opening plenary session of ISOPS VIII, Eunice Galati estimated there are currently 988 valid phlebotomine species and subspecies from all continents except Antarctica, including 29 fossils, with 512 extant and 17 fossil taxa found in the Americas. The genus *Phlebotomus* Rondani & Berté, 1840 has been split and supplemented during the 20th C. Most specialists now accept at least six genera: *Phlebotomus*, *Sergentomyia* França & Parrot, 1920 and *Chinius* Leng, 1987 in the eastern hemisphere; and, *Brumptomyia* França & Parrot, 1921, *Lutzomyia* França, 1924 and *Warileya* Hertig, 1948 in the Americas. However, doubts about the monophyly of the most speciose genera - *Phlebotomus*, *Sergentomyia* and *Lutzomyia* - have been re-inforced by phylogenetic studies based on morphology and morphometry [4-6] as well as by more limited molecular datasets [7], making it increasingly difficult to support the practical classification of Lewis et al. [8], and its modifications [1,9], which place most mammalophilic species and all vectors of human leishmaniasis in the genera *Phlebotomus* (eastern hemisphere) and *Lutzomyia* (Americas) (Table 1).

The phylogenetic analyses of Galati [4,5], based on a non-numerical cladistic approach, identified two tribes: Phlebotomini and Hertigiini. The latter contained two sub-tribes, one from each hemisphere, but only 28 extant species classified in 5 genera and no vectors of human leishmaniasis. In contrast, Phlebotomini was far more speciose, containing 931 extant species classified in 30 genera in six sub-tribes: Phlebotomina (eastern hemisphere), Australophlebotomina (Australia and Asia), Sergentomyiina (both hemispheres), and the exclusively American Brumptomyiina, Lutzomyiina and Psychodopygina. Some of the generic proposals were supported by numerical phylogenetic analyses of faunas from China and the Oriental region [7], and most of the American genera are accepted by many specialists in Latin America and some others.

At ISOPS VIII, Jerôme Depaquit and colleagues (Abstract 18-O; http://www.isops8.org/en/libro-de-resumenes-2/) presented a preliminary molecular phylogenetic analysis of 18S ribosomal DNA and partial 28S ribosomal DNA sequences from a representative range of taxa from all continents. This supported the monophyly of some clades in Galati’s classification [4,5], although others were clearly paraphyletic, including the genus *Phlebotomus* as previously highlighted by Depaquit et al. [10]. Therefore, prospects for expert agreement on a classification of Phlebotominae are much improved, based on a reconciliation of morphological and molecular approaches. This could greatly aid biomedical information retrieval for vector control as well as evolutionary studies on vector-parasite evolution (Table 1). In any new classification, the widespread neotropical vector of visceral leishmaniasis, *Lutzomyia* (*Lutzomyia*) *longipalpis*, might become the type species of a much smaller genus than that considered by Lewis et al. [8], from which most of the American vectors of cutaneous leishmaniasis would be excluded.

### Vector competence

Classically, vector competence was defined by studies on vector-parasite-host interactions, and it should continue to be distinguished from ecological associations (see section on Eco-epidemiology) and transmission dynamics (see Conclusions), which nevertheless are also pertinent to the success or failure of *Leishmania* transmission [1]. Identifying the location of different developmental forms of *Leishmania* in specific parts of the sand fly gut, either by microscopy or molecular markers, is necessary but not sufficient for demonstrating vector competence [11]. Ultimately, demonstration of the presence of infective metacyclic forms of the parasite in the vector’s anterior midgut and experimental transmission of the parasite are central to the characterization of vector competence. Vera Seblova-Hrobarikova and colleagues concluded (Abstract 50-O; http://www.isops8.org/en/libro-de-resumenes-2/) that there is no good experimental evidence for routine development of infective forms of anthropotropic *Leishmania* in *Sergentomyia* species or in ceratopogonid midges [12], although the latter were found naturally infected with a marsupial parasite in Australia [13]. Another important feature of midgut infections is genetic exchange, which may even involve different *Leishmania* species [14]. Hybrids occur naturally, may increase parasite fitness, and therefore could facilitate establishment of disease foci [15].

Other presentations at ISOPS VIII were also pertinent to leishmaniasis control. On the specificity of *Leishmania*-sand fly interactions (Table 1), Paulo Pimenta and colleagues (Abstract 48-O; http://www.isops8.org/en/libro-de-resumenes-2/) reported a glucosylated lipophosphoglycan (LPG) in *Leishmania* (*Leishmania*) *infantum chagasi* strain BH46 that was essential for its early survival but not for midgut attachment, and suggested that other factors such as promastigote secretory gel (PSG) may be more important for establishment in this vector-parasite combination [16]. Tatiana Di-Blasi and colleagues (Abstract 59-O; http://www.isops8.org/en/libro-de-resumenes-2/) also touched on the specificity of such interactions, demonstrating that pre-incubation of *Leishmania* (*Leishmania*) *major* with antibodies against FLAG, a flagellar-specific protein, reduced parasite binding to the midgut of *Phlebotomus* (*Phlebotomus*) *papatasi*, but not of *Le. i. chagasi* to *L. longipalpis*. In “permissive” vectors, like *L. longipalpis*, there is evidence that promastigotes bind to N-acetylgalactosamine-containing glycoconjugates on the sand fly midgut [17].

Concerning modulation of the sand fly innate immune response, André Pitaluga and colleagues (Abstract 54-O; http://www.isops8.org/en/libro-de-resumenes-2/) reported that *Leishmania* infection in *L. longipalpis* caused an early increase in the expression of *cactus*, a repressor of the Toll pathway, accompanied by an early growth of sand fly midgut microbiota. Findings suggest that *Leishmania* may activate a homologue of the mammalian macrophage tyrosine phosphatase (SHP-1) in *L. longipalpis* that in mammals inhibits the Toll and Jak/STAT pathways. Clearly, a complex interaction exists between *Leishmania* and midgut microbiota that has an effect on the development of mature infections [18].

The mechanics of sand fly biting and its influence on the success of transmission was emphasized by Ranadhir Dey and colleagues (Abstract 56-O; http://www.isops8.org/en/libro-de-resumenes-2/), who reported the induction of an acute pro-inflammatory response (IL1-b, IL-6, IFN-g, TNF-a and NOS2) in the skin 3 h after bites infected with *Leishmania* (*Leishmania*) *donovani*. This was followed by a persistent recruitment of neutrophils and alternately activated monocytes at 6-18 h. These early responses, absent from failed needle-initiated infections, contribute to a better understanding of the observed virulence of vector-transmitted leishmaniasis.

Another component of vector transmission is vector saliva, co-deposited with parasites and PSG at the bite site [19]. Building on studies in rodent models of infection, Shaden Kamhawi and colleagues (Abstract 53-O; http://www.isops8.org/en/libro-de-resumenes-2/) showed that immunity to saliva or a salivary molecule protected non-human primates from *Le. major* infection. Protection was associated with the development of a saliva-specific T_H_1-biased delayed-type hypersensitivity response and the accelerated development of a robust *Leishmania*-specific immune response. Wafa Rebai-Kammoun and colleagues (Abstract 57-O; http://www.isops8.org/en/libro-de-resumenes-2/) reported that positive anti-saliva proliferative responses in uninfected individuals living in areas of Tunisia endemic for cutaneous leishmaniasis were associated with protection from *Le. major* infection, reinforcing the conclusion that cellular immunity to vector saliva may protect against *Le. major* infection. Both these studies point to the potential of salivary molecules in *Leishmania* vaccines [20].

An important aspect of vector competence is the efficiency with which a vector species acquires *Leishmania* from infected hosts. Jovana Sadlova and colleagues (Abstract 49-O; http://www.isops8.org/en/libro-de-resumenes-2/) used BALB/c mice to demonstrate that 20% of *Phlebotomus (Larroussius) orientalis* became infected with *Le. donovani* after feeding on inoculated ears or uninoculated contralateral ears, both showing no pathology, but not on other bodyparts. This important finding suggests that asymptomatic individuals may act as reservoirs of the infection, a finding that will have an impact on approaches to vector control. Another interesting study reported by Jan Votypka and colleagues (Abstract 60-O; http://www.isops8.org/en/libro-de-resumenes-2/) indirectly explored the effect of climate change and the expansion of sand fly habitats on the spread of leishmaniasis. *Leishmania (Viannia) braziliensis* and *Le. infantum* were both able to develop in sand flies kept at low temperatures, indicating they could potentially spread with their vectors into new areas with cooler climates [21].

### Genetics, genomics and transcriptomics

The molecular methodologies and analyses that have revolutionized research on mosquito vector biology are only beginning to be fully exploited for sand flies. Molecular markers for sand fly vector identification and incrimination have been applied for c. 30 years, although the potential ubiquity of cryptic species complexes and how they may relate to the transmission of *Leishmania* and disease outcomes is an ongoing debate [1,22]. Markers include mitochondrial genes (e.g. *cytochrome b*, *COI* and *NADH4*) as well as nuclear genes and microsatellite loci (e.g. ribosomal DNA, *elongation factor 1-alpha*) [7]. Some have proved useful for population genetics, but the neutrality of loci has rarely been tested [23]. Vit Dvořák and colleagues (Abstract 20-O; http://www.isops8.org/en/libro-de-resumenes-2/) reported on a relatively new method of molecular identification, namely protein profiling by matrix-assisted laser desorption/ionization time of flight mass spectrometry (MALDI-TOF MS), which has been tested on reference samples of 20 species of *Phlebotomus, Sergentomyia* and *Lutzomyia* [24].

Equally important for developing control measures is the elucidation of the molecular mechanisms that define two-way or three-way interactions between vectors, vertebrate hosts and infectious organisms that include arboviruses as well as *Leishmania*. Molecular genetics and biochemistry have elucidated much about sand fly genes involved in blood meal digestion [25], immune responses [26] and circadian rhythms affecting mating and host-seeking behaviours [27], as well as the parasite-host interactions discussed more fully under vector competence.

There is now an opportunity to advance all these genetics topics, following the recent completion of the first stage of the Sand Fly Genome Sequencing Project, as outlined at ISOPS VIII by Mary Ann McDowell and colleagues (Abstract 61-O; http://www.isops8.org/en/libro-de-resumenes-2/). This project's goal was to sequence the genomes of two sand flies that exhibit distinct distributions, behaviours, and pathogen specificities. The genome of *L. longipalpis* (Jacobina strain) is approximately 150 Mb at 38.9× coverage and the genome of *P. papatasi* (Israeli strain) was sequenced at 22.5× coverage and is approximately 350 Mb. Characterization is nearing completion for gene families involved with G-protein coupled receptors, odorant and gustatory receptors, salivary peptides, hormone signalling, antioxidants, aquaporins, symbiotic interactions, transposable elements and virus transmission. The completed genome assemblies and additional genomic resources can be found on VectorBase (https://www.vectorbase.org; [28]), as explained by Gloria Giraldo-Calderón and colleagues (Abstract 62-O; http://www.isops8.org/en/libro-de-resumenes-2/). Resources include: full transcriptomes and BAC sequences; 40,000 ESTs from a normalized cDNA library of *P. papatasi* generated from the four larval stages, pupae, adult males, unfed females, and females post-feeding on uninfected or *Le. major*-infected mouse blood; and, transcriptomes of *L. longipalpis* for larval stages and adult females fed with sugar and blood meals, uninfected or infected with *Le. infantum*. This project should accelerate the discovery of regulatory and biochemical pathways, vaccine candidates, and targets for insecticide development, sometimes by identifying features unique to sand flies to foster development of novel control technologies.

### Eco-epidemiology

Most presentations (81) at ISOPS VIII concerned the eco-epidemiology of cutaneous and visceral leishmaniasis (Table 1). They had the implicit aim of incriminating sand fly species as vectors, by adding ecological associations to vector competence [1,29]. Many reports investigated links between environmental factors and changes in vector distributions and transmission patterns, which could result from a combination of human activities and natural phenomena such as climate warming (Jeffrey J Shaw, abstract 41-O; http://www.isops8.org/en/libro-de-resumenes-2/). A challenge is to determine whether new environments favour the evolution of new vector phenotypes and genotypes or the selection and emergence of existing ones. There is evidence in São Paulo State, Brazil, that one genotype of *L. longipalpis* is expanding faster than another. Habitat changes also modify reservoir host distributions. As explained by Alon Warburg (Abstract 42-O; http://www.isops8.org/en/libro-de-resumenes-2/), local environment modifications in Israel have favoured the spread of rock hyrax colonies (*Procavia capensis*) close to human habitations, thereby establishing zoonotic transmission of *Leishmania* (*Leishmania*) *tropica*. Other concerns about the emergence of transmission cycles include the potential establishment of *Le. tropica* in Sicily, where the vector *Phlebotomus (Paraphlebotomus) sergenti* is locally abundant (Luigi Gradoni and colleagues, abstract 45-O; http://www.isops8.org/en/libro-de-resumenes-2/) [30].

In ISOPS VIII, natural infections of *Leishmania* were reported for the first time from some sand fly species or regions, adding to the list of potential vectors. For example, in some regions of Colombia the vector of visceral leishmaniasis is *Lutzomyia (Pifanomyia) evansi*, and Alveiro Perez-Doria and colleagues (Abstract 46-O; http://www.isops8.org/en/libro-de-resumenes-2/) found it naturally infected with both *Le. i. chagasi* and *Le. braziliensis*, while Luis R Romero and colleagues (Abstract 51-O; http://www.isops8.org/en/libro-de-resumenes-2/) reported natural infections of *Le.* (*Viannia*) spp. in *Lutzomyia* (*Micropygomyia*) *cayennensis*. Most infections were detected by molecular methods, but it was emphasized that dissection can be just as fast and provides additional important information on the distribution and intensity of the infection in a sand fly [11]. These findings drew attention to the necessity to standardize the use of simple criteria to denote grades of vector status [31], sometimes with the addition of mathematical modelling of transmission [1]. Empirical proof of transmission is critical, but it has only been demonstrated for a small percentage of the species considered to be vectors.

The focality of leishmaniasis transmission is well known, but the determinants are not always understood. In some cases it is considered to be linked to a micro-environment, while in others to an ecosystem [32]. Analyses of the occurrence of visceral leishmaniasis cases in northern Ethiopia (Abstract 34-O; http://www.isops8.org/en/libro-de-resumenes-2/) showed that affected villages occurred inside areas characterized by vertisols, and coincidently the abundance of the incriminated vector, *P. orientalis*, was greater in the same areas. However, within this ecosystem, vector populations were higher in the micro-habitats provided by soil fissures [33].

Feeding preferences can help identify reservoir hosts and the risk of human infection. Increasingly, blood meal sources are identified using PCRs that target *cytochrome b* fragments [34] or the prepronociceptin (PNOC) gene, and such an approach has implicated hares as potential reservoirs of visceral leishmaniais in Spain. Preferred hosts can also be identified by detecting anti-bodies to sand fly salivary peptides [1,35], which was demonstrated for dogs by Tereza Kratochvílová and colleagues (Abstract 73-P; http://www.isops8.org/en/libro-de-resumenes-2/).

### Vector control: Re-assessment of tools

Presentations on vector control were prominent at ISOPS VIII. They included a review of the current preventive measures for leishmaniasis including repellents [1,2] and interventions for canine leishmaniasis [36]. There were reports on the development of old tools, such as indoor residual spraying (IRS) and insecticide-treatment of uniforms and bednets (Albert Picado and colleagues, abstract 1-O; http://www.isops8.org/en/libro-de-resumenes-2/), although there is little base-line data on insecticide resistance [1]. Newer tools include sugar-baited insecticides [37] and systemic insecticides in mammalian hosts [38]. The latter have raised concerns about risks to ecosystems, especially as sand flies and leishmaniasis occur in a diverse range of exotic landscapes.

Increasingly, there is recognition that all vector control strategies should be reassessed and implemented in a framework of decision making and quality assurance that can be applied at the lowest administrative level in a health system [39]. Therefore, the role of health education in the construction of leishmaniasis vector control programmes should be recognized. The success of a control programme can be limited unless the people involved understand the needs for an intervention, their personal participation, and the maintenance of surveillance to prevent the recurrence of transmission.

There is a growing recognition of the need to evaluate the impact of sand fly control tools on clinical outcomes, for which there are few studies [40]. IRS (of DDT) is extensively used to control *Phlebotomus* (*Euphlebotomus*) *argentipes* on the Indian subcontinent, but there is limited scientific evidence to support its efficacy for reducing the incidence of visceral leishmaniasis [40]. In contrast, IRS of alphacypermethrin was associated with reduced incidence of cutaneous leishmanisis in northern Morocco [41]. Using entomological outcomes is informative, but public health recommendations can only be based on results arising from well-designed studies (e.g. randomised controlled trials) that evaluate clinical outcomes in people (e.g. infection, disease incidence). These points are illustrated by a recent cluster randomised controlled trial on the Indian subcontinent, where the use of insecticide treated nets reduced the indoor sand fly density by 25% but did not significantly reduce the risk of *Le. donovani* infection or disease [42].

The reasons explaining such seemingly contradictory results may be complex, and this should prompt a closer collaboration between entomologists working on basic sand fly science (e.g. sand fly behaviour, parasite-vector interactions, vectorial capacity) and those developing and evaluating vector control tools. The need to improve communication between the two groups also applies to veterinary interventions. Many resources have been applied to interrupting the domestic cycle of *Le. infantum* in southern Europe and Latin America [43], but there still needs to be clearer distinctions between measures for the individual protection of pet dogs and interventions for the community protection of domestic dog populations as reservoirs of human visceral leishmaniasis. Information exchange between specialists would help develop more effective control tools as well as translating basic biological research into innovative intervention strategies, such as the development of synthetic sex pheromones in long-lasting lures for *L. longipalpis* in Brazilian foci of visceral leishmaniasis [44].

### Transmission of other pathogens

Phlebotomines are also vectors of arboviruses, including *Phlebovirus* strains causing three-day fevers and Toscana virus causing summer meningitis in the Mediterranean region [1,2,45]; and *Lutzomyia* (*Pifanomyia*) *verrucarum* s.l. is the specific vector of the alphaproteobacterium *Bartonella bacilliformis*, the causative agent of Carrion's disease in Latin America [1,2]. The vectorial transmission of these pathogens is always neglected compared with leishmaniasis, and they were hardly mentioned at ISOPS VIII.

## Conclusions

### Integrated control of leishmaniasis

Research on sand flies as leishmaniasis vectors has revealed a diverse array of transmission cycles (Table 1) associated with much human morbidity and mortality [3]. Cycles include: peri-domestic anthroponoses on the Indian sub-continent (where visceral leishmaniasis caused by *Le. donovani* can result in thousands of deaths annually) and in western Asia (where dry cutaneous “Aleppo boil” caused by *Le. tropica* is rampant in war-torn Afghanistan and Syria); peri-domestic zoonoses in the Mediterranean region and Latin America (where dogs infected with *Le. infantum* are the main reservoirs for human visceral leishmaniasis) and in arid North Africa and Asia (where epidemics of cutaneous disease caused by *Le. major* occur near colonies of the gerbilid rodent reservoirs); as well as the more exotic zoonoses in Latin America [where sloths, anteaters and armadillos are sylvatic reservoirs of *Leishmania* (*Viannia*) *guyanensis*, *Leishmania* (*Viannia*) *panamensis* and related parasites], some of which are emerging peri-domestically [including *Le. braziliensis* and *Leishmania* (*Leishmania*) *amazonensis* found in terrestrial rodents] [1,29,46,47].

The challenge is to separate vectors of biomedical importance from the sand fly species that are competent vectors but lack the vectorial capacity to cause much human disease (Paul Ready, abstract 47-O; http://www.isops8.org/en/libro-de-resumenes-2/). The latter may have ecological associations with infected humans or reservoir hosts, and the descriptive eco-epidemiology might well suggest a potential vectorial role. However, the Entomological Inoculation Rate (EIR) for a *Leishmania* species could still be too low to establish a disease focus. Transmission modelling is required to identify the vectors that are a real public health priority [1,48], the ones that must be controlled as part of the integrated control of leishmaniasis [1,3,47,48], which currently relies on case detection and treatment while human and canine vaccines are in development [46,49,50]. Effective modelling of transmission requires the use of entomological indices such as EIR, ones more precise than those usually reported in the leishmaniasis literature - most articles mention only the relative abundance of all adults of each sand fly species, without even separating the totals of females and non-blood feeding males [1,47]. ISOPS VIII made an important step in the right direction, by placing sand fly vector control centre stage and not as an addendum, where it is often placed at international symposia.
